# Systemic and mucosal humoral immune responses induced by the JY-adjuvanted nasal spray H7N9 vaccine in mice

**DOI:** 10.1038/s41426-018-0133-y

**Published:** 2018-08-03

**Authors:** Jing Xu, Shuxiang Li, Xinyi Wang, Jing Liu, Pu Shan, Ya Zhou, Jing Zhao, Zhibiao Wang, Cui Xu, Meili Chen, Ze Chen, Kai Zhao, Di Qu

**Affiliations:** 1 Key Laboratory of Medical Molecular Virology of MOE and MOH, School of Basic Medical Sciences, Fudan University, Shanghai, China; 2 China National Vaccine and Serum Institute, Beijing, China; 3 Key Laboratory of Pathogenic Microbiology and Immunology, Institute of Microbiology, Chinese Academy of Sciences, Beijing, China; 4 Beijing JDK Bio-Tech Institute, Beijing, China; 5 Dongfang Hospital of Beijing University of Chinese Medicine, Beijing, China; 6 Beijing Bio-Institute Biological Products Co., Ltd., Beijing, China; 7 Shanghai Institute of Biological Products Co., Ltd., Shanghai, China

## Abstract

Since the first case of human avian influenza A (H7N9) virus infection in 2013, five H7N9 epidemics have occurred in China, all of which caused severe diseases, including pneumonia and acute respiratory distress syndrome, and the fatality rates of these epidemics were as high as 30–40%. To control the prevalence of H7N9 influenza, an effective vaccine is urgently needed. In the present study, we used chitosan and recombinant human interleukin-2 as an adjuvant (JY) to promote the systemic and mucosal immune responses induced by the H7N9 vaccine. Mice were immunized intranasally with the inactivated split influenza A (H7N9) virus (A/Shanghai/02/2013) vaccine with or without JY. The hemagglutination inhibition (HI) titers of mice immunized with the JY-adjuvanted vaccine were significantly higher than those of mice immunized with the vaccine without adjuvant (21.11 ± 9.58 vs. 5.04 ± 3, *P* < 0.05). The JY-adjuvanted H7N9 nasal spray vaccine induced higher HI titers (8 ± 0.82 vs. 6.7 ± 0.67, *P* = 0.0035) than those did the poly (I:C)-adjuvanted H7N9 vaccine or the LTB-adjuvanted H7N9 vaccine (8 ± 0.82 vs. 6.9 ± 0.88, *P* = 0.0186). The optimal immunization regimen for the nasal spray H7N9 vaccine was determined to be a 21-day interval between the primary immunization and booster, with a dose of 4.5 μg hemagglutinin per mouse. The immunogenicities of the nasal spray H7N9 vaccine and intramuscular vaccine (containing only the inactivated split virus) were compared in mice. Two doses of the nasal spray H7N9 vaccine induced higher titers of HI (6.7 ± 0.67 vs. 5.3 ± 1.16, *P* = 0.004) and anti-HA IgG in sera (19.26 ± 0.67 vs. 13.97 ± 0.82, *P* < 0.0001) and of anti-HA sIgA (7.13 ± 2.54 vs. 0, *P* = 0.0000) in bronchoalveolar lavage fluid (BALF) than one dose of intramuscular H7N9 vaccine 3 weeks after the last immunization. However, when we immunized the mice with two doses of both vaccines separately, the nasal spray H7N9 vaccine induced higher titers of anti-HA IgG (19.26 ± 0.67 vs. 17.56 ± 0.57, *P* < 0.0001) and anti-HA sIgA (7.13 ± 2.54 vs. 4.02 ± 0.33, *P* = 0.0026) than did the intramuscular H7N9 vaccine, and there was no difference in HI titer between the two groups (*P* = 0.3745). This finding indicates that the JY-adjuvanted nasal spray H7N9 vaccine induced not only the systemic immune response but also a local mucosal response, which may improve the efficacy of H7N9 influenza prevention through respiratory tract transmission.

## Introduction

In March 2013, the avian influenza A (H7N9) virus was isolated from a patient in China, the first case of human infection with the virus worldwide^1^. Most patients infected with H7N9 have a history of exposure to live poultry; however, the virus is not pathogenic to poultry but can cause acute pneumonia in humans. As of 5 September 2017, a total of 1558 laboratory-confirmed human infections with avian influenza A (H7N9) virus had been reported through the International Health Regulations (IHR) notification since early 2013^2^. This severe epidemic prompted the development of the H7N9 vaccine. Hemagglutinin (HA) H7 from inactivated H7N7 influenza vaccine is a poorly immunogenic antigen^3^. In clinical trials, the H7N9 vaccine with AS03 or MF59 adjuvant has been evaluated. In one clinical trial, after two doses of inoculation with the H7N9 vaccine containing 15 μg HA with AS03 or MF59 adjuvant or without an adjuvant, the rate of hemagglutination inhibition (HI) titer ≥40 reached 84% with the AS03 adjuvant, 57% with the MF59 adjuvant, and 2% without adjuvant^4^. In another clinical trial, two doses of AS03-adjuvanted H7N9 vaccine induced 96.2% seroconversion, and the geometric mean titer of HI reached 151.1^5^. It has been suggested that an adjuvant is required for improving the efficacy of the H7N9 intramuscular vaccine. However, the most suitable vaccination route is to imitate the natural microbial infection pathway, such as in the polio oral vaccine. Since the flu is transmitted through the respiratory tract, we tried to develop a nasal spray H7N9 vaccine.

It has been shown that intranasal administration of an influenza vaccine elicits not only a mucosal sIgA response but also a systemic antibody response, as well as a better cellular immune response than intramuscular immunization^6^. FluMist^®^ Quadrivalent (Intranasal Influenza Vaccine) is a cold-adapted, live attenuated influenza vaccine^7^. Medimmune Limited Liability Company has developed a live attenuated H7N9 influenza vaccine using reverse genetics technology. Intranasal immunization with one dose of this recombinant H7N9 strain completely protected ferrets against challenge with the wild-type homologous strain A/Anhui/1/2013 (H7N9) or the heterologous wild-type strain A/Netherlands/219/2003 (H7N7)^8^. Many researchers have attempted to develop effective nasal inactivated mucosal vaccines. Mucosal vaccines face a gauntlet of host defenses as they are diluted in mucosal secretions, captured in mucus gels, attacked by proteases and nucleases, and excluded by epithelial barriers^9^. Therefore, effective adjuvants are needed to protect the antigen and enhance the immune response.

In 2007, Jin-di-ke Biological Research Institute (Beijing, China) developed the JY adjuvant, which is composed of recombinant human interleukin-2 (rhIL-2) and chitosan. The safety of the JY adjuvant has been determined in mice, proving that it is non-irritating and non-toxic^10^. Chitosan is able to increase adhesion to the mucous membrane to promote antigen transport across the mucosal surface^11^ and enhance the uptake of antigens by dendritic cells (DCs) and DC maturation^12,13^. rhIL-2 can activate the Th1 cell immune response^14^.

In this study, we evaluated the immunogenicity of the JY-adjuvanted nasal spray H7N9 vaccine with split virus influenza and determined the optimal immunization interval and dosage of vaccination. We found that the JY-adjuvanted nasal spray H7N9 vaccine can induce a systemic immune response and a local mucosal response.

## Results

### Effect of JY adjuvant on the immunogenicity of nasal spray H7N9 or H1N1 vaccine

**Fig. 1:**
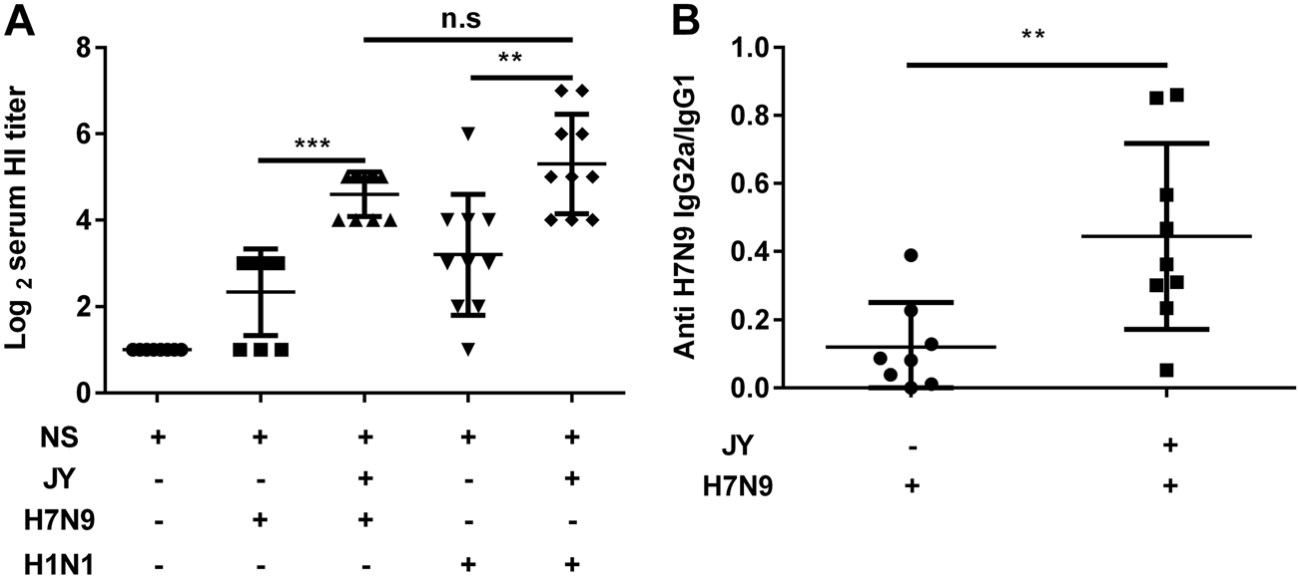
Effect of JY adjuvant on the immunogenicity of the nasal spray H7N9 or H1N1 vaccine. Mice were immunized twice (1-week interval) with the H7N9 or H1N1 influenza vaccine with or without JY adjuvant. The HI titers in serum were determined by the HI assay with virus H7N9 (A/shanghai/02/2013) or H1N1 (A/California/07/2009). The subclasses of the anti-HA IgG (IgG2a and IgG1) in serum were detected by ELISA. The data are shown as the geometric mean of mice in each group with their corresponding SD on a log 2 scale, and the results were compared using Student’s *t*-test. Differences with a *P*-value < 0.05 were considered statistically significant. Significant differences between groups are indicated as **P < 0.01, ***P < 0.001, or n.s. no significant difference

To investigate whether the JY adjuvant was able to enhance the immunogenicity of influenza vaccines, we immunized mice intranasally with the H7N9 or H1N1 influenza vaccine with or without the JY adjuvant. Each of the mice was immunized with 4.5 μg HA twice at a 7-day interval. Serum was collected 2 weeks after the booster. The HI titers of mice immunized with the JY-adjuvanted nasal spray H7N9 vaccine were significantly higher than those of mice immunized with the vaccine without adjuvant (*P* < 0.05) (JY-H7N9 vs. H7N9: 21.11 ± 9.58 and 5.04 ± 3; JY-H1N1 vs. H1N1: 39.39 ± 43.36 and 9.19 ± 18.14). The HI titers of the nasal spray H1N1 vaccine with or without JY adjuvant were slightly higher than those of the H7N9 vaccine, but the difference was not significant (*P* > 0.05) (Fig. 1a). The IgG2a/IgG1 ratio of mice immunized with the JY-adjuvanted nasal spray H7N9 vaccine was significantly higher than that of mice immunized with the non-adjuvant vaccine (0.44 ± 0.21 vs. 0.12 ± 0.13, *P* < 0.05) (Fig. 1b). These findings indicate that JY is able to enhance the immunogenicity of the nasal H7N9 or H1N1 influenza vaccine.

**Fig. 2:**
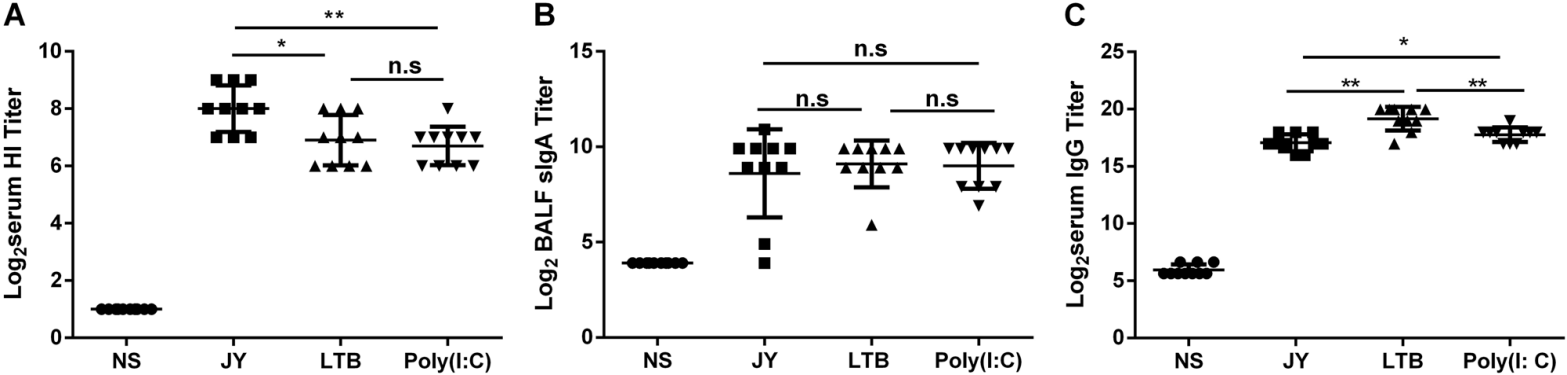
Comparison of the adjuvant effect of different adjuvants on the nasal spray H7N9 vaccine. Mice were intranasally immunized with 4.5 μg HA twice (3-week interval) and with different adjuvants. Serum and BALF were collected at day 21 after the last immunization, and the titers of HI and IgG in serum and of sIgA in BALF were detected. The data are shown as the geometric mean of mice in each group with the corresponding SD on a log 2 scale, and the results were compared using Student’s *t*-test. Differences with a *P*-value < 0.05 were considered statistically significant. Significant differences between groups are indicated as *P < 0.05, **P < 0.01, or n.s. no significant difference

The immune enhancement effect of different adjuvants on the nasal spray H7N9 vaccine was compared. Mice were intranasally immunized with 4.5 μg/mouse HA twice (3-week interval) and with different adjuvants. Serum and BALF were collected at day 21 after the last immunization, and then the titers of HI and IgG in serum and of sIgA in BALF were detected. The JY-adjuvanted H7N9 nasal spray vaccine induced higher HI titers (8 ± 0.82 vs. 6.7 ± 0.67, *P* = 0.0035) than the poly(I:C)-adjuvanted H7N9 vaccine or the LTB-adjuvanted H7N9 vaccine (8 ± 0.82 vs. 6.9 ± 0.88, *P* = 0.0186) (Fig. 2a). There was no difference in the sIgA titer among the three groups (Fig. 2b). The LTB-adjuvanted H7N9 nasal spray vaccine induced higher IgG titers than the other two vaccines (LTB vs. JY: *P* = 0.0011, LTB vs. poly(I:C): *P* = 0.0053) (Fig. 2c).

### Optimization of immunization regimens

**Fig. 3:**
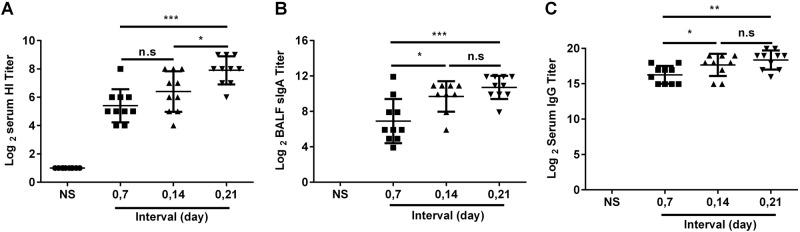
Optimal immunization interval. BALB/c mice were randomly divided into four groups and intranasally immunized with the JY-adjuvanted H7N9 nasal spray vaccine containing 4.5 μg HA at 7-day, 14-day, and 21-day intervals or an NS control. Three weeks after the last immunization, serum and BALF were collected. The titers of HI and anti-HA IgG in serum and of sIgA in BALF were detected using the HI assay or ELISA. The data are shown as the geometric mean of all mice in each group with the corresponding SD on a log 2 scale, and the results were compared using Student’s *t*-test. Differences with a *P*-value < 0.05 were considered statistically significant. Significant differences between groups are indicated as *P < 0.05, **P < 0.01, ***P < 0.001, or n.s. no significant difference

It was important to determine the optimal immunization regimen of the JY-adjuvanted nasal spray H7N9 vaccine. At first, we investigated the interval between doses of the nasal spray H7N9 vaccine, and mice were immunized intranasally at 7-day, 14-day, and 21-day intervals. Serum and BALF were collected 21 days after the second immunization. The titers of HI and anti-HA IgG in serum, as well as of anti-HA sIgA in BALF, were detected using the HI assay or ELISA. In the 21-day interval group, the HI titers (7.9 ± 0.74 vs. 5.4 ± 0.88, *P* = 0.0001) (Fig. 3a), anti-HA IgG titers (10.71 ± 1.04 vs. 6.91 ± 2, *P* = 0.0020) (Fig. 3c), and sIgA titers (18.37 ± 1.12 vs. 16.27 ± 1.1, *P* = 0.0005) (Fig. 3b) were significantly higher than those in the 7-day interval group, while the HI titers in the 21-day interval group were significantly higher than those in the 14-day interval group (7.9 ± 0.74 vs. 6.4 ± 1.2, *P* = 0.0139) (Fig. 3a). Thus, the 21-day interval was used in subsequent experiments.

**Fig. 4:**
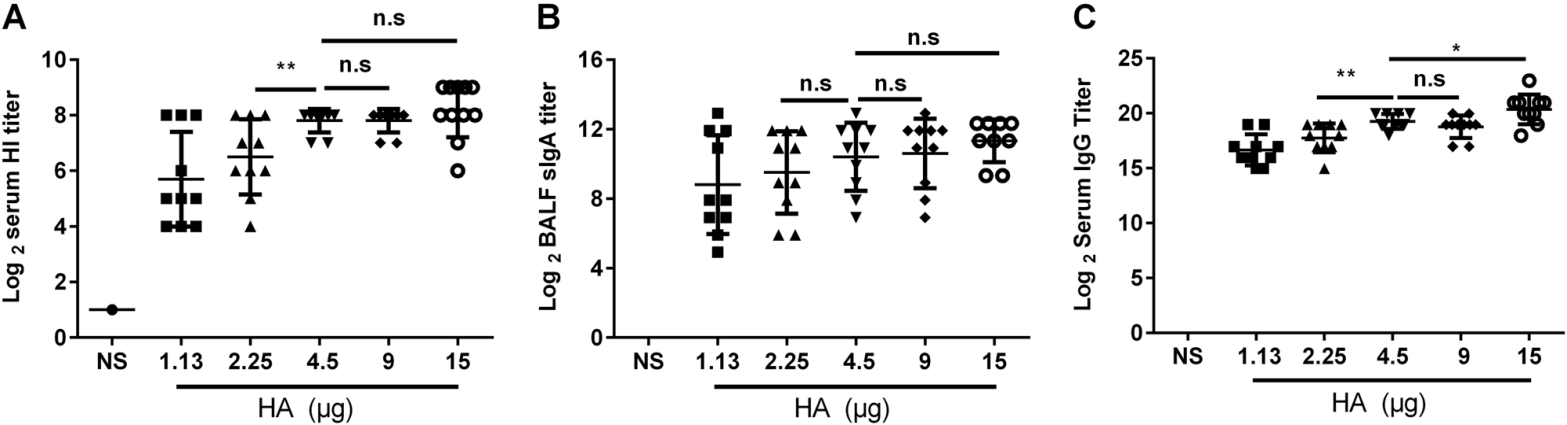
Optimal dose selection. BALB/c mice were randomly divided into five groups and intranasally immunized with the JY-adjuvanted nasal spray H7N9 vaccine with a 21-day interval but with different HA contents: 15, 9, 4.5, 2.25, or 1.13 μg. An NS control group was also included. Serum and BALF were collected 21 days after the last immunization. The titers of HI and anti-HA IgG in serum and of sIgA in BALF were detected using the HI assay or ELISA. The data are shown as the geometric mean of all mice in each group with the corresponding SD on a log 2 scale, and the results were compared using Student’s *t*-test. Differences with a *P*-value < 0.05 were considered statistically significant. Significant differences between groups are indicated as *P < 0.05, **P < 0.01, or n.s. no significant difference

Once we determined the optimal dosage of vaccine, mice were immunized intranasally with different doses (each mouse was immunized with 15, 9, 4.5, 2.25, or 1.13 μg HA) of the nasal spray H7N9 vaccine and boosted at a 21-day interval. Serum and BALF were collected 21 days after the second immunization. The titers of HI (Fig. 4a) and anti-HA IgG (Fig. 4c) in the mice immunized with 4.5 μg HA were significantly higher than those in the mice immunized with 2.25 μg HA (*P* = 0.0096, *P* = 0.0049) and 1.13 μg HA (*P* = 0.0014, *P* < 0.0001). The anti-HA IgG titers in mice immunized with 15 μg HA were significantly higher than those in mice immunized with 4.5 μg HA (*P* = 0.0333), but there was no difference in the titers of sIgA and HI (*P* > 0.05) between the two groups (Fig. 4b). Thus, 4.5 μg HA/mouse was used in the following vaccination schedule.

### Immunogenicity comparison of the nasal spray H7N9 vaccine and intramuscular vaccine

**Fig. 5:**
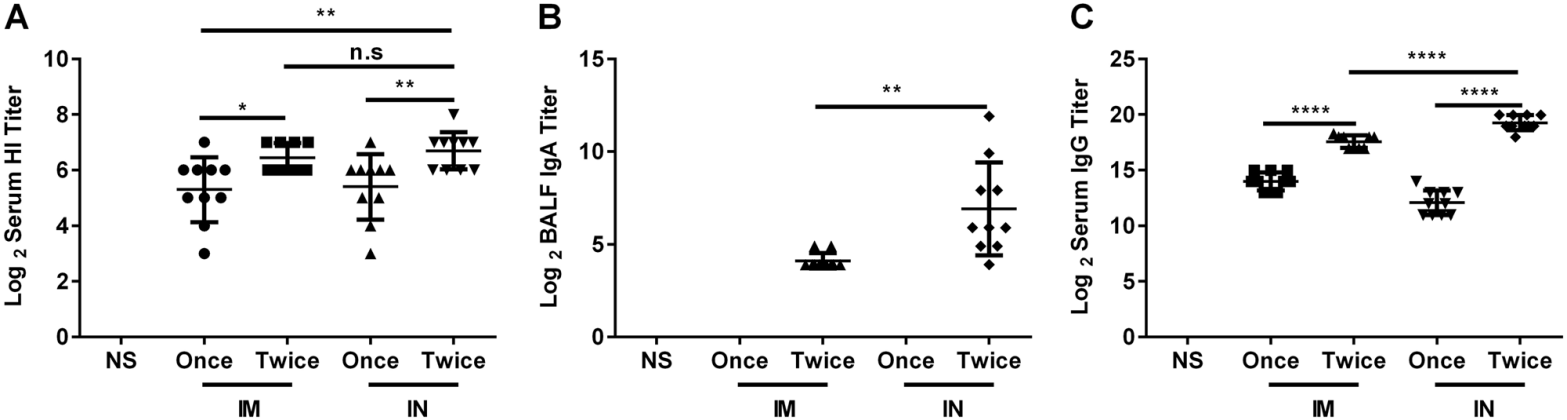
Immunogenicity comparison of the nasal spray H7N9 vaccine and the intramuscular vaccine. Mice were intranasally or intramuscularly immunized with vaccines once or twice (3-week interval). Serum and BALF were collected 21 days after the last immunization, and then the titers of HI and anti-HA IgG in serum and of sIgA in BALF were determined. The data are shown as the geometric mean of all mice in each group with the corresponding SD on a log 2 scale, and the results were compared using Student’s *t*-test. Differences with a *P*-value < 0.05 were considered statistically significant. Significant differences between groups are indicated as *P < 0.05, **P < 0.01, ****P < 0.0001, or n.s. no significant difference. IN intranasal administration, IM intramuscular administration

The immunogenicities of the nasal spray and intramuscular H7N9 vaccine were compared at the optimal immunization interval (21 days) and dosage (4.5 μg HA/mouse) (Fig. 5). Mice were immunized intranasally or intramuscularly with vaccines once or twice (21-day interval). Serum and BALF were collected 21 days after the last immunization. Two doses of nasal spray H7N9 vaccine immunization induced higher HI titers (6.7 ± 0.67 vs. 5.4 ± 1.17, *P* = 0.007), sIgA titers (7.13 ± 2.54 vs. 0, *P* = 0.0000), and anti-HA IgG titers (19.26 ± 0.67 vs. 12.07 ± 1.10, *P* < 0.0001) than a single dose. Two doses of nasal spray H7N9 vaccine immunization induced higher HI titers (6.7 ± 0.67 vs. 5.3 ± 1.16, *P* = 0.004), sIgA titers (7.13 ± 2.54 vs. 0, *P* = 0.0000), and anti-HA IgG titers (19.26 ± 0.67 vs. 13.97 ± 0.82, *P* < 0.0001) than one dose of the intramuscular H7N9 vaccine. Two doses of nasal spray H7N9 vaccine immunization induced higher anti-HA IgG titers (19.26 ± 0.67 vs. 17.56 ± 0.57, *P* < 0.0001) and anti-HA sIgA titers (7.13 ± 2.54 vs. 4.02 ± 0.33, *P* = 0.0026) than two doses of the intramuscular H7N9 vaccine. There was no difference in HI titer between the two groups (*P* = 0.3745) (Fig. 5).

## Discussion

Since the first case of human avian influenza A (H7N9) virus infection in 2013, five H7N9 epidemics have occurred in China. As of 5 September 2017, a total of 1558 laboratory-confirmed human infections with avian influenza A (H7N9) virus have been reported through the IHR notification since early 2013^2^. The clinical data of Madan et al. indicated that immunization with two doses of AS03-adjuvanted H7N9 vaccine can achieve a 96% seroconversion rate, but there was no sIgA response in the local mucosal membrane^5^. Most viral and bacterial infections are acquired through mucosal membranes. The nasal route offers a promising opportunity for the delivery of vaccines. Intranasal administration is simple, convenient, and painless. It does not require intrusive injections, such as with needles, avoids the risk of cross-contamination and local irritation, and increases public acceptance^15^. However, nasal immunization with a vaccine must overcome some limitations because the nasal cilia removal efficiency affects the retention time of the antigen in the nasal mucosa, and the nasal mucus pH affects the stability of the antigen.

The live attenuated vaccine FluMist^®^ was approved in 2003; during its application for more than 10 years, the efficacy of influenza prevention has been significant. In 2016, the CDC advised discontinuing the use of FluMist^®^ in the US; for the past three flu seasons, the vaccine seemed to have little efficacy. In the UK, however, the vaccine was still recommended. The discrepancy in efficacy data between the US and the UK suggested that an intranasal vaccine may still face significant challenges. In addition to FluMist^®^, which harbored cold-adapted strains, NasalFlu was an inactivated nasal spray influenza vaccine adjuvanted with heat labile toxin (HLT)^16^, which caused 46 cases of temporary facial paralysis (Bell’s palsy) in the 7 months after its approval^17^. NasalFlu was recalled because of the side effect of the HLT adjuvant. Thus, it is essential to choose a safe and effective adjuvant.

In the present study, we used a novel mucosal adjuvant-JY to develop the nasal spray H7N9 vaccine. The JY adjuvant is composed of chitosan and IL-2, which does not have neurotoxicity like HLT and has a certain advantage in terms of safety. We used H7N9 and H1N1 split virus-prepared nasal spray vaccines with JY adjuvant and evaluated the immunogenicity. HI titers of mice immunized with JY-adjuvanted nasal spray H7N9 or H1N1 vaccine were significantly higher than those of mice immunized with the vaccine without the adjuvant. There was no difference between the two subtypes of nasal spray vaccines. These data suggested that the JY adjuvant can enhance the immunogenicity of the nasal spray H7N9 influenza vaccine.

The JY adjuvant is composed of chitosan and IL-2. Chitosan can act on the tight junction ZO-1 protein and cytoskeletal actin in the intercellular matrix. After blocking ZO-1 and cytoskeletal actin, the intercellular tight junction opens, promoting antigen transport and uptake by antigen-presenting cells, such as DCs and macrophages, thereby stimulating the production of cytokines and enhancing the adaptive immune response^18–20^. Th0 cells transform into Th1 or Th2 cells in response to stimulation by different antigens, cytokines, or antigen-presenting cells. Th1 cells can effectively stimulate and mediate cellular immune responses, stimulate cytotoxic T cells, and activate macrophages, as well as play an important role in clearing intracellular bacteria. These cells provide an auxiliary function for B cells to produce IgG2a subtype virus-neutralizing antibodies in mice. Th2 cells stimulate the humoral immune response, and secretion of IL-4 can promote B cell proliferation and induce antibody production, in which the production of IgA and antibacterial toxin-neutralizing IgG antibody plays a supporting role. Compared with the non-adjuvant group, the JY-adjuvanted group had significantly higher levels of IgG1 and IgG2a. The IgG2a/IgG1 ratio of the JY-adjuvanted nasal spray H7N9 vaccine group was significantly higher than that of the non-adjuvant group. These results demonstrated that the JY adjuvant not only enhanced antigen immunogenicity but also promoted Th1 responses, which are beneficial for virus clearance and disease recovery. The ratio of IgG2a/IgG1 can characterize the Th1/Th2 immune responses, as reported in many studies^21–23^. Indeed, in recent years, some studies have characterized the Th1 and Th2 immune responses by combined detection of specific cytokines secreted by SPL and the IgG2a/IgG1 ratio^24,25^. We will detect cytokines secreted by SPL to characterize the Th1 and Th2 immune responses in the future.

We optimized the immunization regimens for the nasal spray H7N9 vaccine and found that each mouse immunized with 4.5 μg HA twice at a 21-day interval could induce a strong immune response. Because of the barrier function of the mucosal system, it is difficult to obtain good immunity with a single inoculation. A booster immunization is needed to further improve the immune response. FluMist (Quadrivalent) was also administered twice over 1 month in children who were not immunized with the influenza vaccine at the age of 2–8 years. The inactivated influenza intranasal vaccine NasalFlu also required a double inoculation.

Most of the approved influenza vaccines are intramuscular inoculations without adjuvant. We would like to know whether the JY-adjuvanted nasal spray H7N9 vaccine can induce a local mucosal immune response (sIgA) and whether its induced serum HI titers can reach the levels induced by intramuscular injection of influenza vaccines. The immunogenicity of the JY-adjuvanted nasal spray H7N9 vaccine and the intramuscular vaccine (without adjuvant) were compared. Two doses of the JY-adjuvanted nasal spray H7N9 vaccine induced higher HI and anti-HA IgG titers in serum, as well as higher sIgA titers in BALF, than those induced by one dose of the traditional vaccine administered intramuscularly. Compared with two doses of intramuscular vaccination, the JY-adjuvanted vaccine resulted in higher IgG titers in serum and higher sIgA titers in BALF. In this study, HI titers induced by the JY-adjuvanted nasal spray H7N9 vaccine were comparable to those induced by the intramuscular vaccine, which is consistent with findings for other mucosal vaccine immunizations^26^. However, it can induce a sIgA response in the local mucosal membrane. Two doses of JY-adjuvanted nasal spray H7N9 vaccine induced higher sIgA titers than did one or two doses of intramuscular H7N9 vaccine (*P* < 0.0001). This mucosal IgA response may provide protection in local tissues by inhibiting virus replication and spread in the respiratory tract. We also evaluated serum IgA levels. Two doses of nasal spray H7N9 vaccine immunization induced higher serum IgA titers than one dose of intramuscular H7N9 vaccine (*P* < 0.001), while there was no difference between two doses of the nasal spray H7N9 vaccine and the intramuscular H7N9 vaccine (Supplementary Data).

In summary, we have shown that the JY adjuvant is a useful mucosal adjuvant to induce mucosal and systemic responses against mucosal H7N9 infection in mice. The administration of the JY-adjuvanted nasal spray H7N9 vaccine induced not only a systemic immune response but also a local mucosal response. In the future, we shall investigate the protection against lethal challenge with H7N9 influenza viruses in mice or ferrets offered by the nasal spray H7N9 vaccine. In addition, we shall investigate adjuvant only, as well as immunized nasal spray vaccine groups, to ensure that the protection is not through adjuvant-directed innate responses.

## Materials and methods

### Preparation of influenza split virus vaccines

The influenza H7N9 split virus vaccine was produced by Beijing Tiantan Biological Products Co., Ltd. (Beijing, China), and the influenza H1N1 split virus vaccine was produced by Zhejiang Tianyuan Biological Pharmaceutical Co., Ltd. (Hangzhou, China). The vaccine was prepared in 10-day-old embryonated chicken eggs using techniques identical to those employed in the current production of the trivalent split vaccine against seasonal influenza.

### Preparation of JY-adjuvanted nasal spray influenza vaccines

For the preparation of the rhIL-2 stock solution, 1,000,000 IU of rhIL-2 (Changchun Institute of Biological Products Co., Ltd., Changchun, China) was dissolved in 1 ml normal saline. For the preparation of the 1% (w/v) chitosan stock solution, 1 g chitosan (Heppe Medical Chitosan GmbH, Halle, Germany) was dissolved in 100 ml 0.4% acetic acid solution and stirred at 200 rpm for 2 h. The chitosan was dissolved completely, filtered with a 0.45 μm filter membrane and stored at 2–8 °C. Next, the H7N9 or H1N1 split virus vaccine stock solution and rhIL-2 solution were added directly to the chitosan solution. The final concentrations of chitosan, rhIL-2, and HA were 0.1% (w/v), 100,000 U/ml, and 225 μg/ml, respectively. The vaccines were stored at 2–8 °C prior to use.

### Mouse immunization

#### Mice

Female BALB/c mice (6–8 weeks old) were provided by Beijing Weitong Lihua Experimental Animal Technology Co., Ltd. (Beijing, China) and provided sterilized food and water under specific pathogen-free conditions. All animal experiments were approved by and conducted in accordance with the guidelines of the Animal Research Ethics Committee of the China National Vaccine and Serum Institute. Ten mice were included in each group in all experiments.

### Immunization of mice with JY-adjuvanted nasal spray H7N9 influenza vaccines

Mice were randomly divided into five groups: the H7N9 (A/Shanghai/02/2013) or H1N1 (A/California/07/2009) influenza vaccine with or without JY adjuvant groups and the NS control group. Each of the mice was intranasally immunized with 4.5 μg HA twice at 7-day intervals^16^. Serum was collected 2 weeks after the booster, and HI titers in the serum were determined. The IgG subclass (IgG2a and IgG1) of the nasal spray H7N9 vaccine was detected, and the ratio of IgG2a/IgG1 was determined.

We further compared the adjuvant effect of JY adjuvant with that of LTB and poly(I:C). Mice were immunized with nasal spray H7N9 influenza with JY adjuvant, LTB or poly(I:C) (InvivoGen, San Diego, CA, USA) (Table 1). Serum and BALF were collected at day 21 after the last immunization, and then the titers of HI and IgG in the serum and of sIgA in the BALF were determined.

**Table 1:** Intranasal immunization design in the different groups

Groups	*n*	Time	HA/mouse	Adjuvant/mouse
Control	10	Days 0, 21	−/NS	−/NS
JY	10	Days 0, 21	4.5 μg	10 μl
LTB	10	Days 0, 21	4.5 μg	5 μg
Poly(I:C)	10	Days 0, 21	4.5 μg	5 μg

### Optimization of vaccination regimens

To study the effects of different immunization intervals on the immune response of the JY-adjuvanted nasal spray H7N9 vaccine, we immunized mice at 7-day, 14-day, and 21-day intervals. Three groups of mice were anesthetized with 0.5 ml of 0.25% pentobarbital sodium by intraperitoneal injection and then intranasally immunized with the JY-adjuvanted nasal spray H7N9 vaccine with 4.5 μg HA. The control group was intranasally immunized with NS at days 0 and 14. Serum and BALF were collected 21 days after the second immunization. The titers of serum HI, anti-HA IgG, and BALF sIgA were detected.

To study the effects of different dosages on the immune response to the JY-adjuvanted nasal spray H7N9 vaccine, we immunized mice with different doses of HA: 15, 9, 4.5, 2.25, or 1.13 μg. Six groups of mice were used. Groups 1, 2, 3, 4, and 5 were immunized twice at days 0 and 21 (3-week interval) with different doses, and group 6 was immunized with NS as the control group. Serum and BALF were collected 21 days after the second immunization. The titers of serum HI, anti-HA IgG, and sIgA in BALF were detected.

### Comparison of the immunogenicity of the nasal spray H7N9 vaccine and intramuscular injection H7N9 vaccine

The immunogenicities of the JY-adjuvanted nasal spray H7N9 vaccine and the intramuscular injection split vaccine were compared. Mice were randomly divided into five groups. Groups 1 and 2 were intranasally immunized with 4.5 μg HA once or twice (3-week interval), while groups 3 and 4 were intramuscularly injected with 4.5 μg HA once or twice (3-week interval). Group 5 was the control group immunized with NS. Serum and BALF were collected 21 days after the last immunization, and the titers of HI and anti-HA IgG in serum and sIgA in BALF were determined.

### Preparation of bronchoalveolar lavage fluid

Mice were euthanized, the thoracic cavity was opened, and the trachea was exposed and infused with 0.3 ml NS to aspirate BALF^27^. BALF was collected and centrifuged at 1500 rpm for 5 min, and the supernatant was stored at −20 °C prior to the assays.

### HI assay

Serum preparation and the experiment were carried out according to the reference method^28–30^. First, 50 μl of 1% chicken red blood cell suspension (collected from Hang chickens, Beijing Tainan Biological Products Co., Ltd., Beijing, China) was added to each well, and the plates were incubated at room temperature (20–25 °C) for 30 min and observed at a 45° angle. Additionally, a virus control (serum-free) and erythrocyte control (serum-free and virus added) were prepared. The HI titer of the serum was the maximum dilution that completely inhibited erythrocyte agglutination.

### Detection of specific IgG in serum and sIgA in BALF

The titers of serum anti-HA IgG and BALF sIgA were detected as described by Takahashi et al^31^. For the detection of anti-HA IgG in serum from experiments in which mice were immunized with the H7N9 vaccine, the H7N9 influenza virus split vaccine was coated onto a 96-well plate at 100 μl/well (HA 1 μg/ml, determined by single radial immunodiffusion assay) and incubated at 2–8 °C overnight. To detect BALF sIgA, the H7N9 influenza virus split vaccine was coated onto a 96-well plate at 100 μl/well (HA, 5 μg/ml) and incubated at 2–8 °C overnight. The plates were blocked with 2% bovine serum albumin for 1 h. After the plates were washed with 1‰ Tween 20-PBS washing buffer, a 2-fold serial dilution of mouse serum was added to the plate, which was incubated at 37 °C for 1 h. Next, a 1:20,000 dilution of horseradish peroxidase (HRP)-labeled goat anti-mouse IgG (Thermo Fisher Scientific, Waltham, MA, USA) or a 1:5000 dilution of HRP-labeled goat anti-mouse IgAα chain (Abcam, Cambridge, UK) was added, and the plates were incubated at 37 °C for 1 h. A tetramethyl benzidine (TMB) solution was added, followed by incubation at 37 °C for 10 min. After the stop solution was added, the absorbance values of the plate wells were read in a microplate reader (Multiskan MK3, Thermo, San Jose, CA, USA) at 450/630 nm. The cut-off value was 2.1 times the absorbance of the NS control group; for example, if the absorbance of the NS control group was less than 0.05, then the cut-off value was 0.105. Values higher than the cut-off value were considered positive. The sample titer was determined by the highest dilution factor with an absorbance reading higher than the cut-off.

### Detection of specific IgG subclasses

We used a kit (Mouse IgG1/IgG2a ELISA Quantitation Set; Bethyl Laboratories, Inc., Montgomery, TX, USA) to detect the subclasses of anti-HA IgG in serum from immunized mice. First, a goat anti-mouse IgG1-coating or IgG2a-coating antibody was diluted at 1:100 and added to 96-well plates, followed by incubation at room temperature for 1 h. The split H7N9 vaccine (A/Shanghai/02/2013) was diluted to 1 μg/ml (HA content, as determined by the single radial immunodiffusion assay) and added to a 96-well plate at 100 μl/well, followed by incubation at 37 °C for 2 h. Plates were washed five times with 0.05% Tween 20 wash solution, 200 μl of blocking solution (0.05 M Tris, 0.14 M NaCl, 1% BSA) was added, and the plates were incubated for 30 min at room temperature. IgG1 or IgG2a standards in the kit were diluted according to the instructions. After the samples were diluted 1:1000, a 2-fold dilution series was prepared (dilution buffer: 0.05 M Tris, 0.14 M NaCl, 0.05% Tween 20, 1% BSA). Next, 100 μl/well of the standards and serum samples were incubated at room temperature for 1 h. After washing, 100 μl/well of HRP-labeled anti-IgG1 (1:100,000 dilution) or IgG2a antibody (1:50,000 dilution) was added to the plates, which were incubated at room temperature for 1 h. TMB solution was added, and the plates were incubated at 37 °C for another 15 min. After adding stop solution, the absorbance values were read at 450 nm. A standard curve was prepared to determine the amount of mouse IgG1 or IgG2a in the samples. The average absorbance value minus the blank value for each standard concentration was plotted on the vertical (*Y*) axis vs. the corresponding mouse IgG1 or IgG2a concentration on the horizontal (*X*) axis using curve-fitting software. The serum IgG2a/IgG1 ratio was calculated for each group.

### Statistical analysis

Experimental data were analyzed using SPSS software and were compared using Student’s *t*-test. Differences with a *P*-value < 0.05 were considered statistically significant. Graphs were plotted using GraphPad Prism 6 (GraphPad Software, La Jolla, CA, USA).

## Electronic supplementary material

Supplementary Data
