# Supplementary material for: Systemic and mucosal humoral immune responses induced by the JY-adjuvanted nasal spray H7N9 vaccine in mice
Source: Emerg Microbes Infect. 2018 Aug 3;7:140. doi: 10.1038/s41426-018-0133-y (PMC6076272; doi:10.1038/s41426-018-0133-y)
Supplement: Supplementary file 1 — Supplementary Data [file 41426_2018_133_MOESM1_ESM.doc]

Supplementary Data:

FigureS1. Immunogenicity comparison of the nasal spray H7N9 vaccine and intramuscular vaccine. Mice were intranasally or intramuscularly immunized with vaccines once or twice (3-week interval). Serum was collected 21 days after the last immunization, and then, the titers of IgA in serum was determined. The data are shown as the geometric means of mice per group with the corresponding SD on a log 2 scale and were compared using Student’s *t*-test. Differences with a 𝑃 value <0.05 were considered statistically significant. Significant differences between groups are indicated, **P* < 0.05. ****P* < 0.001. Ns: no significant difference. IN: intranasal administration, IM: intramuscular administration.
